# Diversity and seasonal abundance of *Culicoides* (Diptera, Ceratopogonidae) in Shizong County, Yunnan Province, China

**DOI:** 10.1051/parasite/2022027

**Published:** 2022-05-11

**Authors:** Ying Liang Duan, Glenn Bellis, Bing Gang Liu, Le Li

**Affiliations:** 1 Yunnan Tropical and Subtropical Animal Virus Diseases Laboratory, Yunnan Animal Science and Veterinary Institute Kunming 650224 Yunnan Province China; 2 Research Institute for the Environment and Livelihoods, Charles Darwin University Darwin NT 0909 Australia; 3 Department of Agriculture, Water and the Environment Darwin NT 0820 Australia; 4 Center for Animal Disease Control and Prevention 651200 Lufeng Yunnan Province China

**Keywords:** *Culicoides*, Vector, Seasonal abundance, BTV, Yunnan, China

## Abstract

*Culicoides* (Diptera, Ceratopogonidae) are small biting midges, some of which are vectors for animal associated arboviruses such as bluetongue virus (BTV) and Akabane virus (AKAV). BTV and AKAV are both pathogenic for livestock, with BTV in particular posing a major threat to domestic ruminants. Ongoing problems with BTV in ruminants in Shizong County of Yunnan Province, China, promoted a year-long investigation of the *Culicoides* in Shizong to determine relative abundance and seasonality of midges and to attempt to identify species that might be acting as vectors in the area. *Culicoides* were collected by UV light trap for one night per week for most weeks between May 2020 and May 2021. More than 21,000 specimens consisting of at least 21 species belonging to six subgenera and one unplaced group, including 5 species previously associated with BTV and one associated with AKAV, were collected. *Culicoides tainanus* dominated most collections throughout the year although *C. sumatrae* was often the dominant species over summer. Most species were abundant between May and October. These results indicate that *C. tainanus, C. jacobsoni* and *C. oxystoma* are the major midge pests of livestock in Shizong and should be considered in any disease investigation.

## Abstract (fr)

Les culicoïdes (Diptera, Ceratopogonidae) sont de petits moucherons piqueurs, dont certains sont des vecteurs d’arbovirus associés aux animaux tels que le virus de la fièvre catarrhale du mouton (FCMV) et le virus Akabane (AKAV). Le FCMV et l’AKAV sont tous deux pathogènes pour le bétail, le FCMV en particulier constituant une menace majeure pour les ruminants domestiques. Les problèmes persistants de FCMV chez les ruminants dans le comté de Shizong de la province du Yunnan, en Chine, ont encouragé une enquête d’un an sur les *Culicoides* à Shizong pour déterminer l’abondance relative et la saisonnalité des culicoïdes et tenter d’identifier les espèces qui pourraient agir comme vecteurs dans la région. Les culicoïdes ont été collectés par piège à lumière UV une nuit par semaine pendant la plupart des semaines entre mai 2020 et mai 2021. Plus de 21 000 spécimens comprenant au moins 21 espèces appartenant à six sous-genres et un groupe non placé, dont 5 espèces précédemment associées au FCMV et une associée à l’AKAV, ont été collectés. *Culicoides tainanus* a dominé la plupart des collectes tout au long de l’année bien que *C. sumatrae* ait souvent été l’espèce dominante pendant l’été. La plupart des espèces étaient abondantes entre mai et octobre. Ces résultats indiquent que *C. tainanus*, *C. jacobsoni* et *C. oxystoma* sont les principaux culicoïdes nuisibles pour le bétail à Shizong et devraient être pris en compte dans toute enquête épidémiologique.

## Introduction

*Culicoides* (Diptera: Ceratopogonidae) are small biting midges. More than 1300 species are known [6] and several of these are vectors of arboviruses, protozoa and nematodes [24, 38]. At least 40 species are associated with the transmission of around 50 arboviruses belonging to three families; the Peribunyaviridae (formerly Bunyaviridae [13]), Reoviridae, and Rhabdoviridae [25]. Furthermore, species of *Culicoides* are the sole vector for approximately 45% of these viruses, which include economically important viruses such as bluetongue virus (BTV), African horse sickness virus (AHSV), epizootic hemorrhagic disease virus (EHDV), and Akabane virus (AKAV) [25].

Of these midge-borne viruses, BTV is the most economically important. The first case of clinical Bluetongue disease (BT) in China was reported in sheep in Shizong County of Yunnan Province in 1979 [15, 39, 40]. Subsequently, BTV has been discovered in approximately half of all provinces in China, including Hubei (1983), Anhui (1985), Guangxi (1985), Sichuan (1988), Shanxi (1993), Guangdong, Jilin, Liaoning, Xinjiang and Tibet [15, 36] and is now regarded as one of the most common livestock-associated Orbiviruses in the country [15, 21, 40].

Studies on the vector potential of Chinese *Culicoides* for livestock arboviruses have tended to focus on detection of virus in wild-caught specimens (for example Duan *et al.* and Di *et al.*) [7, 8, 10, 11]. While this information only satisfies one of the four criteria required to prove the vector status of a species [32, 33], it can be used to screen large numbers of species to determine candidate species for the more detailed laboratory-based studies required to satisfy the vector infection and transmission capacity criteria. The fourth criterion, an accumulation of epidemiological data associating an insect species with the host of the pathogen, can additionally be used to screen large numbers of species to determine candidate species for further study. Important epidemiological data include the abundance, host range and seasonality of a species but until recently, few studies addressing these factors have been conducted in China. Liu *et al.* [18] and Di *et al.* [7] reported the relative abundance of *Culicoides* on livestock farms in border areas of Yunnan province, while Liu *et al.* [19] reported similar data from Jiangxi province. Neither study, however, reported on the seasonality of species or correlated these with the seasonality of virus prevalence.

Following the BT epidemics in the area, viral activity has been monitored using sentinel herds at Wulong village in Shizong County, Yunnan Province from 1995 to 1997 [15] and 2012 to 2016 [26, 36]. Although there have been no outbreaks of BT in sheep in Shizong since 1979, the virus has been silently circulating in cattle and goats with seropositive rates ranging from 13% to 60% [26] and at least nine serotypes (BTV-1, 2, 3, 4, 5, 9, 12, 16, and 24) being isolated [15, 26, 36, 37]. The background data on viral prevalence and seasonality generated during these studies provided an opportunity to investigate the potential of various species of *Culicoides* in Shizong to act as vectors of BTV and other midge-borne viruses at this site.

## Materials and methods

### *Culicoides spp*. collection

**Figure 1:**
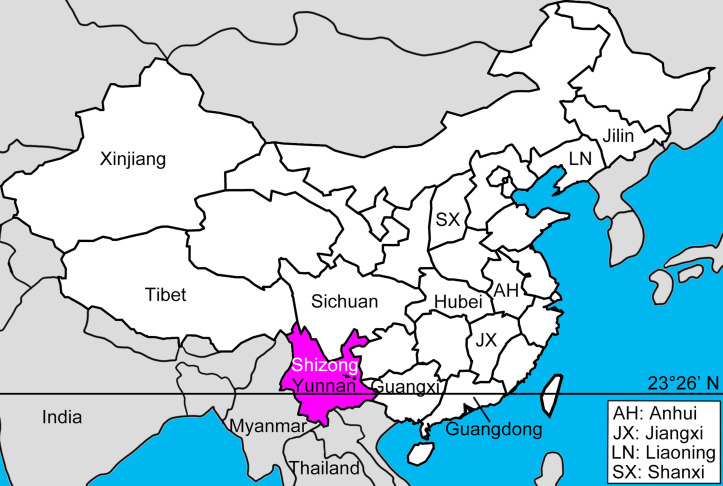
Sketch Map. The collection site (Shizong) and BTV associated provinces, as well as some neighbouring countries are labeled.

Midges were collected for one night per week for most weeks between May 2020 and May 2021, except an 8-week period between 15 January and 20 March 2021. Collections were made in Wulong Village (24°38′24″ N, 104°17′24″ E), Shizong County, Yunnan Province (Fig. 1) using a battery-powered UV light trap (LTS-M02, Wuhan Lucky Star Medical Treatment Technology Co., Wuhan, China). Traps were set inside a cattle shed, approximately 4 m from 4 penned cattle run from 5 pm to 9 am the following day. There were no other livestock in the vicinity of the cattle shed. Midges were collected either into PBS buffer (May–November 2020) and transferred into 70% ethanol within 48 h, or directly into 70% ethanol (December 2020– May 2021).

### Specimen mounting and morphologic identification

*Culicoides* were sorted into species by wing pattern and gross morphology [3, 17, 34] and counted. Representative specimens of each species were mounted following Bellis *et al.* [5] except that wings were removed and mounted onto glass microscope slides, while the remainder of the insect was cleared in 10% KOH overnight, prior to dehydration in ethanol, then clove oil and mounted onto the same slide as the wings.

*Culicoides* species were identified using the keys of Yu *et al.* [17], Wirth & Hubert [34] and Bellis [3]. Subgeneric placement of species follows the system proposed by Wirth & Hubert [34].

### Data analysis

Total specimens of each species were counted and where needed, collated into monthly averages. A heatmap of weekly totals was constructed by the R programming language [12, 20, 41] using the pheatmap package [16]. Rainfall and temperature data for Shizong County of Yunnan Province between May 2020 and May 2021 were gleaned from the Chinese historic weather website [1]. Rainfall was classified into five categories based on the volume of rain over a 24-hour period. These were rainstorm (50.0–99.9 mm), heavy rain (25.0–49.9 mm), moderate rain (10.0–24.9 mm), and light rain (0.1–9.9 mm) or overcast [2] and the number of days per month meeting each of these rainfall categories were recorded for each month.

## Results

### Species diversity

**Figure 2:**
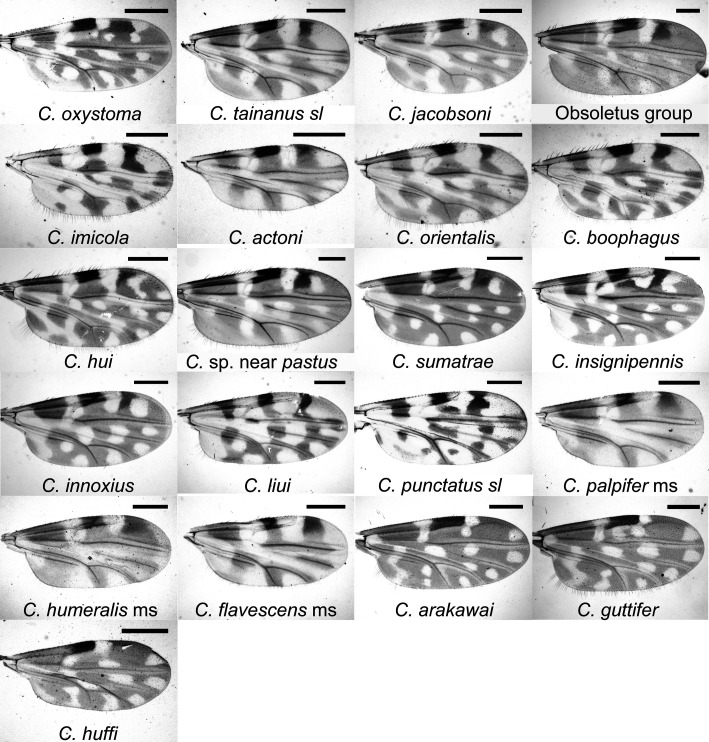
Photographs of representative female wings of the 21 *Culicoides* species collected at Wulong, Shizong County, Yunnan Province, China. Scale bar length = 250 μm.

More than 21,000 specimens of *Culicoides* were collected. At least 21 species belonging to six subgenera (*Avaritia* Fox, *Culicoides* Latreille, *Hoffmania* Fox, *Meijerehelea* Wirth & Hubert, *Remmia* Glukhova, and *Trithecoides* Wirth & Hubert) and one unplaced species group (*Clavipalpis* group) were identified by morphology (Table 1). The wing patterns of the 21 identified species, species groups and morphospecies are shown in Figure 2.

**Table 1:** Abundance of *Culicoides* species collected in UV traps at Wulong, Shizong, Yunnan Province, China between May 2020 and May 2021.

Species	Subgenus	Total number (proportion)	Number of Ranked first a
*C. actoni* Smith	*Avaritia*	47 (0.2%)	0
*C. arakawae* Arakawa	*Meijerehelea*	981 (4.7%)	3
*C. boophagus* Macfie	*Avaritia*	46 (0.2%)	0
*C. flavescens* ms	*Trithecoides*	78 (0.4%)	0
*C. guttifer* de Meijere	*Meijerehelea*	52 (0.2%)	0
*C. huffi* Causey	*Clavipalpis* group	4 (0.0%)	0
*C. hui* Wirth & Hubert	*Avaritia*	7 (0.0%)	0
*C. imicola* Kieffer	*Avaritia*	49 (0.2%)	0
*C. innoxius* Sen & Das Gupta	*Hoffmania*	209 (1.0%)	0
*C. insignipennis* Macfie	*Hoffmania*	561 (2.7%)	0
*C. jacobsoni* Macfie	*Avaritia*	1,046 (5.0%)	2
*C. liui* Wirth & Hubert	*Hoffmania*	157 (0.7%)	0
Obsoletus group	*Avaritia*	321 (1.5%)	1 b
*C. orientalis* Macfie	*Avaritia*	864 (4.1%)	0
*C. oxystoma* Kieffer	*Remmia*	1,559 (7.4%)	0
*C. palpifer* ms	*Trithecoides*	1,424 (6.8%)	1
*C. humeralis* ms	*Trithecoides*	152 (0.7%)	0
*C.* species near *C. pastus*	*Avaritia*	17 (0.1%)	0
*C. punctatus s.l.*	*Culicoides*	109 (0.5%)	0
*C. sumatrae* Macfie	*Hoffmania*	6,214 (29.5%)	9
*C. tainanus s.l.*	*Avaritia*	7,096 (33.7%)	17
Uncertain species		63 (0.3%)	0
Total		21,056 (100%)	

a Number of collections dominated by this species.

b Equal numbers of *C. obsoletus* group and *C. arakawae* in this collection.

Specimens belonging to *C.* subgenus *Trithecoides* are difficult to identify unless mounted and it was impractical to mount all specimens, so they have been grouped into morphospecies that resemble described species. For example, specimens with an entirely yellow scutum and leg banding consistent with *C. palpifer* have been classified as *C. palpifer* morphospecies, abbreviated *C. palpifer* ms. Similarly, specimens with a yellow scutum and leg banding consistent with *C. flavescens* have been classified as *C. flavescens* ms, and specimens with dark brown markings along the anterior margin of the scutum have been classified as *C. humeralis* ms. (Table 1).

Female specimens of the Obsoletus group of *C.* subg. *Avaritia* are difficult to separate and Yunnan populations have been shown to have distinct genetic makeup to populations of existing species [8] so remain unidentified. As such, we have listed our specimens to the lowest taxonomic unit that we can confidently refer them to, which is the Obsoletus group. A similar situation exists with oriental populations of *C. punctatus* which have historically been difficult to place into a species [31], display a high degree of morphological variation, and differ genetically from European populations [22]*.* In light of this uncertainty, we refer our specimens to *C. punctatus sensu lato.* Additionally, Duan *et al.* [8, 10] reported the existence of potentially several cryptic species within *C. tainanus* populations in Yunnan but as we are unable to distinguish these morphologically, they are reported here under *C. tainanus sensu lato*.

**Figure S1:**
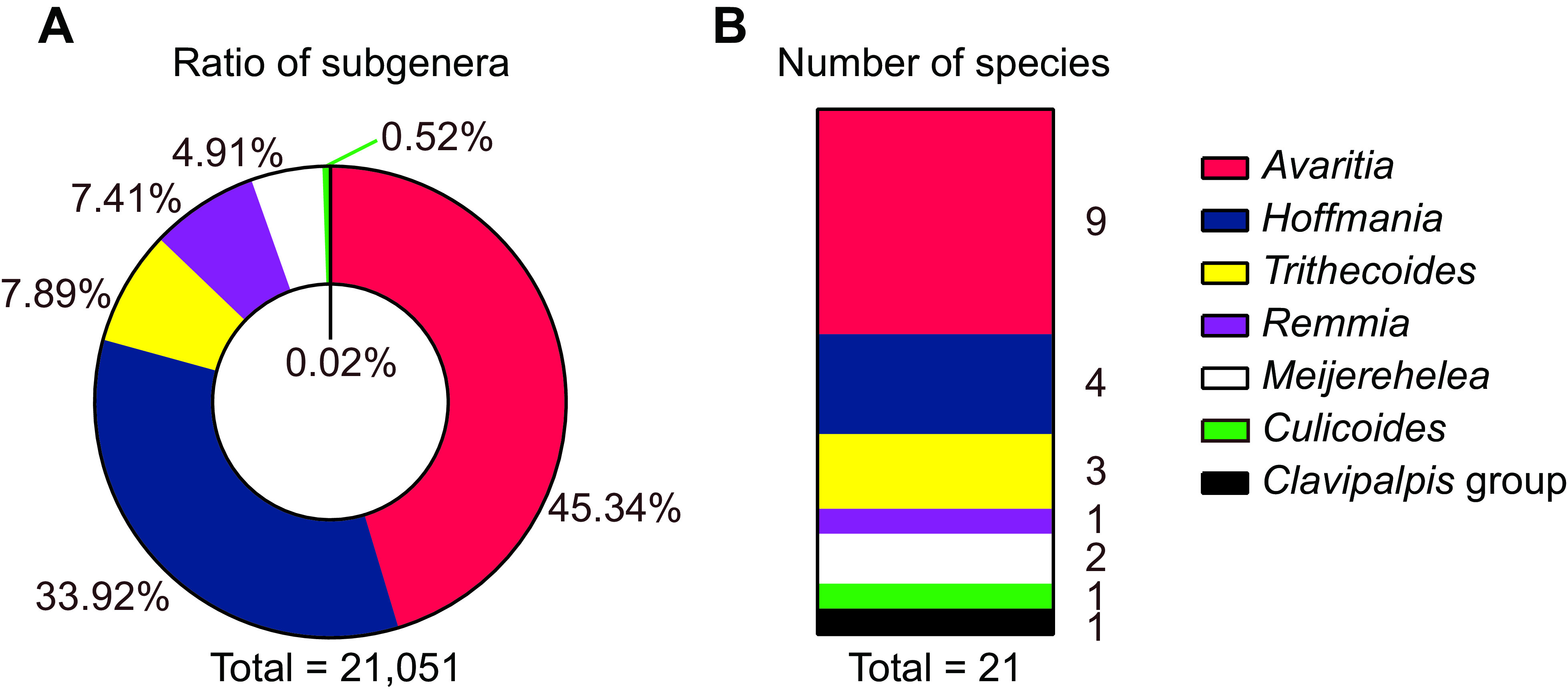
Diversity and relative abundance of specimens belonging to subgenera collected at Wulong between 2020 and 2021. (A) Proportion of *Culicoides* specimens belonging to species from different subgenera or groups; (B) number of species belonging to each subgenus.

The bulk of specimens from our collections belonged to species from *C.* Subg. *Avaritia* (45.34%) and *C.* Subg. *Hoffmania* (33.92%); the remaining specimens belonged to species from *C.* Subg. *Trithecoides* (7.89%), *C.* Subg. *Remmia* (7.41%) and *C.* Subg. *Meijerehelea* (4.91%), and the *Clavipalpis* group (Fig. S1-A). Three of these subgenera *C.* Subg. *Avaritia*, *C.* Subg. *Hoffmania*, and *C.* Subg. *Trithecoides* were represented by nine, four, and three species, respectively (Fig. S1-B).

During this study the dominant species were *C. tainanus s.l.* (33.7%), followed by *C. sumatrae* (29.5%), *C. oxystoma* (7.4%), *C. palpifer* ms (6.8%) and *C. jacobsoni* (5.0%) (Table 1). These results are mostly supported by the number of collections in which a species is dominant except that *C. oxystoma* was never the dominant species and several of the smaller collections were dominated by *C. arakawai* and the Obsoletus group (Table 1)*.*

### Seasonal and relative abundance

**Figure 3:**
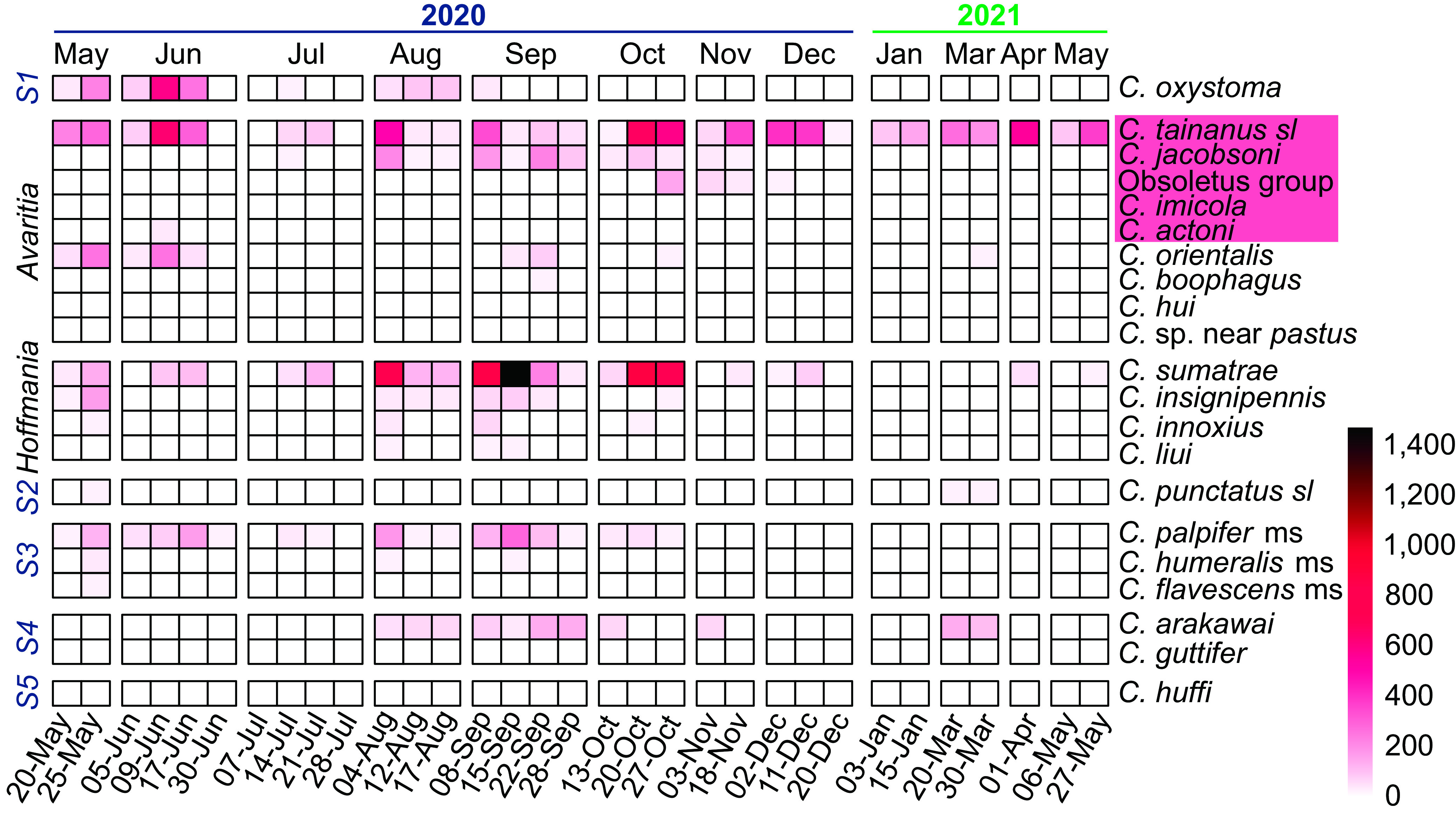
Heatmap of *Culicoides* abundance in collections from Wulong, Shizong County, Yunnan Province, China between May 20, 2020 and May 27, 2021. Known or suspected vectors of BTV are highlighted in red. *S1*, *S2*, *S3*, *S4* and *S5* represent the *Culicoides* subgenera *Remmia*, *Culicoides*, *Trithecoides*, *Meijerehelea*, and the *Clavipalpis* group, respectively.

The numbers of each of the 21 species and species groups from each collection are shown in a heatmap (Fig. 3). Species previously associated with BTV are highlighted in red. The abundance of most species peaked between May and October except for July 2020, although *C. tainanus s.l.* appeared to be active in most months and *C. arakawai* showed a small peak in March (Fig. 3). *Culicoides oxystoma* was most active between May and June, while *C. sumatrae* and *C. jacobsoni* were most abundant between August and October (Fig. 3).

**Figure 4:**
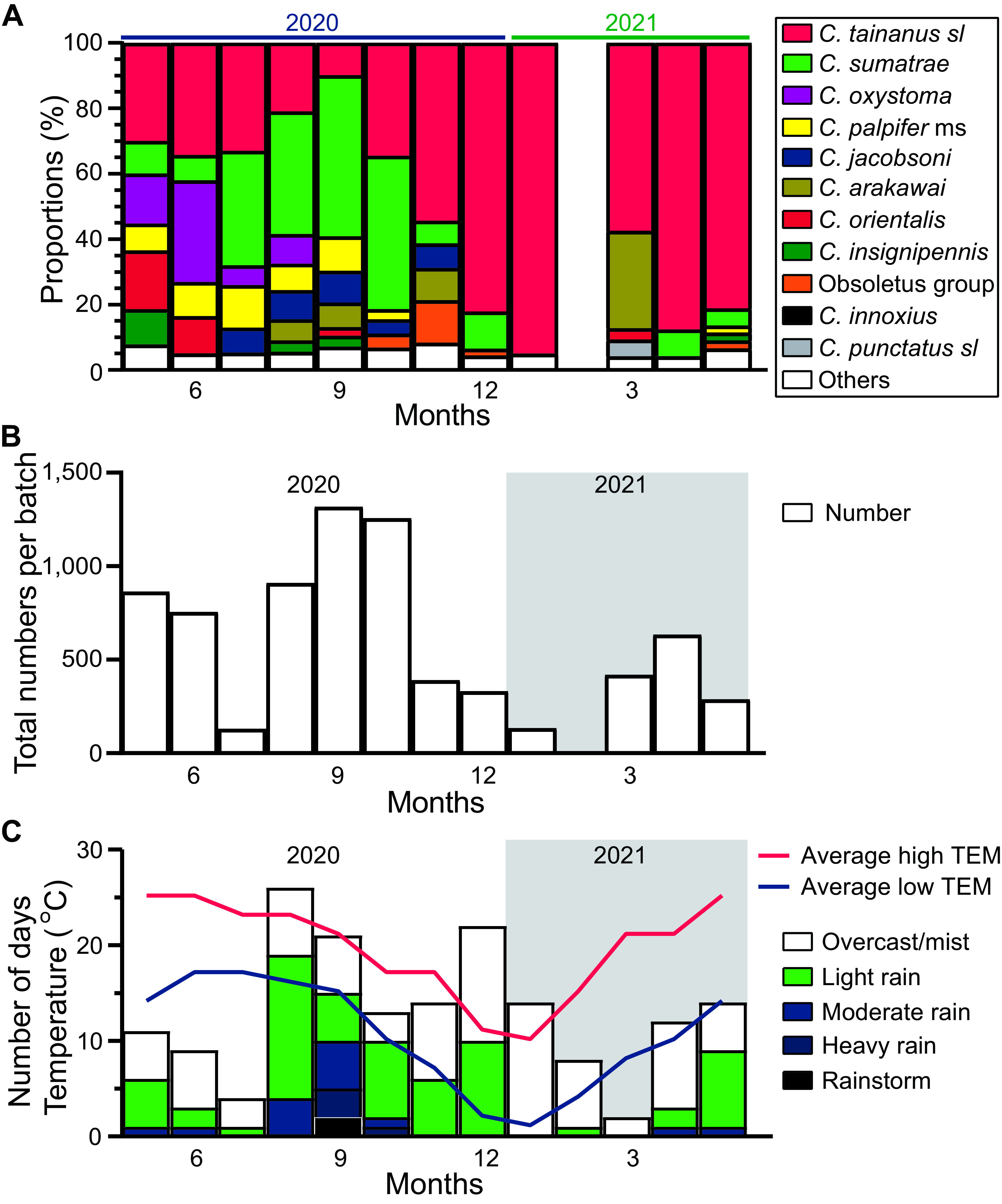
Seasonal abundance of *Culicoides*. A) Relative monthly abundance of *Culicoides* at Wulong, Shizong County, Yunnan Province, China between May 2020 and May 2021. Note that no trapping was done between 15 Jan and 20 Mar 2021; B) average *Culicoides* amount of each batch of collection every month; and C) average monthly maximum temperatures (TEM, red line) and minimum temperatures (blue line) and the number of the days per month experiencing one of the five precipitation categories. The period in 2021 is highlighted in a grey background.

Relative abundance of species varied between seasons with *C. sumatrae* dominating most collections over summer (between July and October) and *C. tainanus s.l.* dominating most collections for the rest of the year (Fig. 4A). Monthly average totals of *Culicoides* per batch of collection suggested that *Culicoides* were active during the summer and autumn except for July 2020, and reached the peak in September (Fig. 4B). The low numbers in July coincided with a period of hot, dry weather (Fig. 4C).

### Potential BTV vectors in Shizong

**Figure 5:**
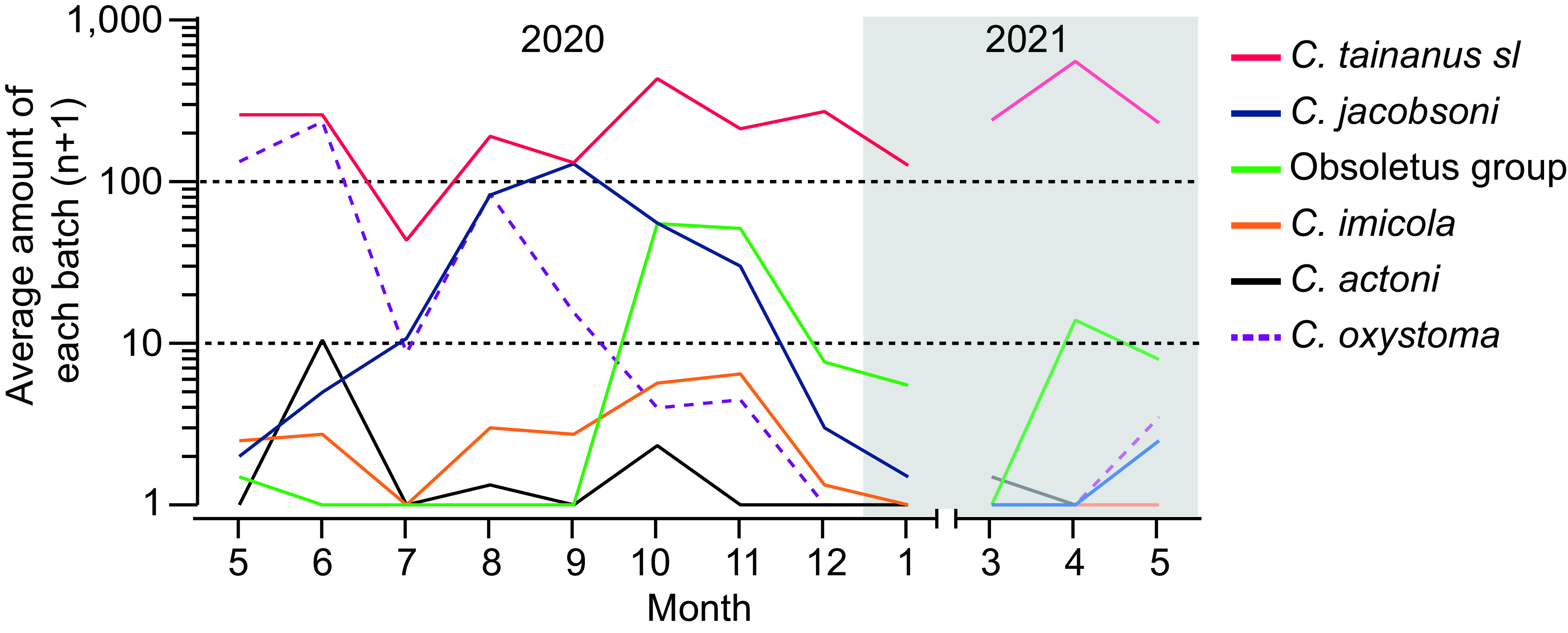
Average monthly numbers of potential BTV vector species at Wulong, Shizong, Yunnan Province, China between May 2020 and May 2021. Logarithm axe and midge amounts (*n* + 1) are shown on the *y*-axis. The period in 2021 is highlighted in a grey background.

Six species previously associated with BTV (*C. tainanus s.l.*, *C. jacobsoni*, the Obsoletus group, *C. imicola*, and *C. actoni*) or AKA (*C. oxystoma*) were collected during this study. Of these, only *C. tainanus s.l., C. jacobsoni* and *C. oxystoma* were present in large numbers, although specimens of the Obsoletus group did dominate one collection in November (Figs. 3, 5). As mentioned above, *C. tainanus s.l.* was present throughout the year and dominated most collections, while *C. jacobsoni* was only active between August and November and the Obsoletus group mainly appeared in November (Fig. 5). *Culicoides oxystoma* was active between May and August, except for the dry July in 2020.

## Discussion

The relative abundance of *Culicoides* on the Wulong cattle farm differs markedly from that recently reported in border areas of Yunnan by Di *et al.* [7] who reported *C. oxystoma* and *C. nipponensis* as the most abundant and widespread species, but the latter species was not collected at all at Shizong. Conversely, four of the five most dominant species at Shizong, *C. tainanus s.l., C. jacobsoni, C. sumatrae* and species belonging to *C.* subg. *Trithecoides* were not reported at any of the five sites (Hekou, Ruili, Mangshi, Longchuan, and Tengchong) studied by Di *et al.* [7]. The sites sampled by Di *et al.* [7] are approximately 300–650 km from Shizong and while their sites included pig farms, bovid farms were also sampled. They also used a different trap to ours although both traps used UV as the attractant. Such variable species diversity and relative abundance within a single province makes it difficult to extrapolate results across studies. Yunnan has very diverse ecological zones ranging from tropical lowland rainforest in the southeast to alpine temperate climates in the northwest, so variation in species composition across the province is not unexpected.

It is unusual that very few *Culicoides* of any species were collected during late June and July, but this may be attributed to unseasonal dry weather at the time (Fig. 4). Immature *Culicoides* spp. require moist habitats and the lack of moisture could have contributed to the low population numbers over this period [28, 29].

Insect vectors of arbovirus are infected when feeding on an infected host and transmit the virus to vertebrate hosts through subsequent feeding, and the maintenance of viral transmission in the field is closely associated with the abundance of vectors [28, 35]. Furthermore, the vectors should be confirmed by an association between vector, host and epidemiology of the virus [32, 33]. Here we have documented species diversity and relative abundance of *Culicoides* species in UV trap collections at a cattle shed in Shizong, Yunnan Province. Based on this data, it would appear that the most prevalent species attacking cattle in this county are *C. tainanus s.l., C. sumatrae, C. oxystoma, C. palpifer* ms and *C. jacobsoni*. Among these, *C. tainanus s.l.,* and *C. jacobsoni* have been associated with BTV and *Tibet Orbivirus* (TIBOV) [8, 10, 11, 14], and *C. oxystoma* might also be infected by BTV [7], but there is no evidence associating any *Orbiviruses* with either *C. sumatrae* and *C. palpifer* ms [10, 11, 14]. The abundance of these species in the cattle shed at Wulong suggests that further investigation of the vector status of these species is warranted.

Only two of the species (*C. imicola* and *C. actoni*) collected in Shizong are proven vectors of BTV according to the four criteria described by WHO [32, 33], but neither of these species were common, comprising only 0.2% of the species present (Table 1). However, *C. actoni* is known to be active prior to sunset [4], therefore the population of *C. actoni* may be underestimated by the UV trapping after dusk.

Prevalence data for BTV in Shizong County indicates that the virus is active in livestock between May and October [26, 36, 40]. This period did not coincide with the peak activity of *C. tainanus s.l.* suggesting that this species may not be as important to the epidemiology of BTV as the other species. However, although *C. tainanus s.l.* was relatively active during all seasons, low temperature will prolong the stages of *Culicoides* life cycle and reduce frequency of biting [28, 35]. Low temperatures also block viral replication in *Culicoides* [28], because the RNA-dependent RNA polymerase of BTV is inhibited below 10 °C [30]. *Culicoides tainanus s.l.* may, however, play a role as a vector, since despite low temperatures during spring and winter potentially reducing the ability of this species to replicate virus, transmission has been observed in sheep in February 1980 in Shizong [39]. With continuing changes in climate, the importance and distribution of vector species like *C. tainanus s.l.* may change as well.

So far, the mechanism of BTV over-wintering is unknown. Ruminants are considered as amplifying hosts of BTV during winter [28], but research [9, 26, 36, 40] indicates that BTV only persists in cattle, sheep, and goats for 2–3 months, which does not explain the absence of BTV between November and April in Shizong [26, 36]. It is unknown if progeny of vectors can be vertically infected, Osborne *et al.* [27] failed to prove vertical transmission of BTV. The presence of healthy populations of *C. tainanus s.l.* throughout the year raises the possibility that this species may be maintaining viral transmission at low levels throughout the winter. Alternatively, the longevity of *C. tainanus s.l.* is unknown but may extend to several months in cold temperatures which would then introduce the possibility of the virus being carried between seasons in infected adult *C. tainanus s.l.*, as observed in *C. sonorensis* [23].

## Supplementary material

The supplementary material of this article is available at https://www.parasite-journal.org/10.1051/parasite/2022027/olm.

## References

[1] Anonymous. 2021. Historic weather of Shizong. [cited 3 Aug 2021]; Available from: https://lishi.tianqi.com/shizong/202001.html.

[2] Anonymous. 2021. Chinese web for popularization of meteorology. [cited 3 Aug 2021]; Available from: http://www.qxkp.net/.

[3] Bellis GA. 2020. Key to females of economically important species of *Culicoides* subgenus *Avaritia* from southern Asia and Australasia using characters visible under a stereomicroscope. [cited 24 Apr 2020]; Available from: https://www.gnatwork.ac.uk/sites/gnatwork/files/content/attachments/2020-04-24/Economic%20Avaritia%20Key.pdf.

[4] Bellis GA, Melville LF, Hunt NT, Hearnden MN. 2004. Temporal activity of biting midges (Diptera: Ceratopogonidae) on cattle near Darwin, Northern Territory. Australia. Veterinaria Italiana, 40(3), 324–328. 20419687

[5] Bellis GA, Dyce AL, Gopurenko D, Mitchell A. 2013. Revision of the Immaculatus Group of *Culicoides* Latreille (Diptera: Ceratopogonidae) from the Australasian Region with descriptions of two new species. Zootaxa, 3680(1), 15–37.

[6] Borkent A, Dominiak P. 2020. Catalog of the biting midges of the World (Diptera: Ceratopogonidae). Zootaxa, 4787, 1–377. 10.11646/zootaxa.4787.1.1 33056456

[7] Di D, Li C, Li Z, Wang X, Xia Q, Mona S, Li B, Liu K, Shao D, Qiu Y, Soe-soe W, Yang S, Wei J, Ma Z. 2021. Detection of arboviruses in *Culicoides* (Diptera: Ceratopogonidae) collected from animal farms in the border areas of Yunnan Province, China. Journal of Integrative Agriculture, 20(9), 2491–2501.

[8] Duan YL, Bellis G, Li L, Li HC, Miao HS, Kou ML, Liao F, Wang Z, Gao L, Li JZ. 2019. Potential vectors of bluetongue virus in high altitude areas of Yunnan Province, China. Parasites & Vectors, 12(1), 464. 31585545 10.1186/s13071-019-3736-9 PMC6778386

[9] Duan YL, Miao HS, Liao F, Kou ML, Li ZH, Wang Z, Li HC, Li L. 2019. The serologic investigation and viral isolation of bluetongue virus in Shangri-La in Southwest China. Transboundary and Emerging Disease, 66(6), 2353–2361. 10.1111/tbed.13292 PMC6899809 31298817

[10] Duan YL, Li L, Bellis G, Yang ZX, Li HC. 2021. Detection of bluetongue virus in *Culicoides* spp. in southern Yunnan Province, China. Parasites & Vectors, 14(1), 68. 33482882 10.1186/s13071-020-04518-z PMC7821528

[11] Duan YL, Yang ZX, Bellis G, Li L. 2021. Isolation of *Tibet Orbivirus* from *Culicoides jacobsoni* (Diptera, Ceratopogonidae) in China. Parasites & Vectors, 14(1), 432. 34454575 10.1186/s13071-021-04899-9 PMC8401062

[12] Greene CS, Tan J, Ung M, Moore JH, Cheng C. 2014. Big data bioinformatics. Journal of Cellular Physiology, 229(12), 1896–1900. 24799088 10.1002/jcp.24662 PMC5604462

[13] ICTV. 2019. ICTV Taxonomy history: *Peribunyaviridae*. Taxonomy History 2016 [cited 21 Feb 2022]; Available from: https://talk.ictvonline.org/taxonomy/p/taxonomy-history?taxnode_id=202000081

[14] Kato T, Shirafuji H, Tanaka S, Sato M, Yamakawa M, Tsuda T, Yanase T. 2016. Bovine arboviruses in *Culicoides* biting midges and sentinel cattle in Southern Japan from 2003 to 2013. Transboundary and Emerging Disease, 63(6), e160–e172. 10.1111/tbed.12324 25597441

[15] Kirkland PD, Zhang N, Hawkes RA, Li Z, Zhang F, Davis RJ, Sanders DA, Li H, Zhang K, Ben J, He GF, Hornitzky CL, Hunt NT. 2002. Studies on the epidemiology of bluetongue virus in China. Epidemiology & Infection, 128(2), 257–263. 12002544 10.1017/s0950268801006525 PMC2869819

[16] Kolde R. 2015. pheatmap: Pretty Heatmaps. [cited 31 Aug 2021]; Available from: http://cran.nexr.com/web/packages/pheatmap/index.html.

[17] Liu JH, Liu GP, Liu ZJ, Yan G, Hao BS, Zhao TS, Yu YX. 2005. Ceratopogonidae: Culicoides, in Ceratopogonidae of China. Yu YX, Editor. Military Medical Science Press: Beijing. p. 816–1323.

[18] Liu GP, Guo XF, Li YY, Zhang J, Wang J, Li CM, Yang ZM, Chen HY, Zhou HN, Liang GD. 2016. Survey of hematophagous midges in China-Laos border. Chinese Journal of Vector Biology and Control, 27(5), 463–466.

[19] Liu Y, Tao H, Yu Y, Yue L, Xia W, Zheng W, Ma H, Liu X, Chen H. 2018. Molecular differentiation and species composition of genus *Culicoides* biting midges (Diptera: Ceratopogonidae) in different habitats in southern China. Veterinary Parasitology, 254, 49–57. 29657011 10.1016/j.vetpar.2018.02.035

[20] Lortie CJ, Braun J, Filazzola A, Miguel F. 2020. A checklist for choosing between R packages in ecology and evolution. Ecology and Evolution, 10(3), 1098–1105. 32076500 10.1002/ece3.5970 PMC7029065

[21] Maclachlan NJ. 2011. Bluetongue: history, global epidemiology, and pathogenesis. Preventive Veterinary Medicine, 102(2), 107–111. 21570141 10.1016/j.prevetmed.2011.04.005

[22] Matsumoto Y, Yanase T, Tsuda T, Noda H. 2009. Characterization of internal transcribed spacer (ITS1)-ITS2 region of ribosomal RNA gene from 25 species of *Culicoides* biting midges (Diptera: Ceratopogonidae) in Japan. Journal of Medical Entomology, 46(5), 1099–1108. 19769041 10.1603/033.046.0517

[23] Mayo CE, Mullens BA, Reisen WK, Osborne CJ, Gibbs EP, Gardner IA, MacLachlan NJ. 2014. Seasonal and interseasonal dynamics of bluetongue virus infection of dairy cattle and *Culicoides sonorensis* midges in northern California-implications for virus overwintering in temperate zones. PLoS One, 9(9), e106975. 25215598 10.1371/journal.pone.0106975 PMC4162562

[24] Meiswinkel R, Venter GJ, Nevill EM. 2004. Vectors: *Culicoides* spp., in Infectious diseases of livestock, Coetzer JAW, Tustin RC, Editors. Oxford University Press: Oxford. p. 93–136.

[25] Mellor PS, Boorman J, Baylis M. 2000. *Culicoides* biting midges: their role as arbovirus vectors. Annual Review of Entomology, 45, 307–340. 10.1146/annurev.ento.45.1.307 10761580

[26] Meng JX, He YW, Xiao L, Li N, Song JL, Wang JL, Li HC. 2018. Dynamic monitoring and infection on bluetongue virus in cattle and goats in Shizong County, Yunnan. Chinese Journal of Zoonoses, 34(6), 537–541.

[27] Osborne CJ, Mayo CE, Mullens BA, McDermott EG, Gerry AC, Reisen WK, MacLachlan NJ. 2015. Lack of evidence for laboratory and natural vertical transmission of Bluetongue Virus in *Culicoides sonorensis* (Diptera: Ceratopogonidae). Journal of Medical Entomology, 52(2), 274–277. 26336312 10.1093/jme/tju063 PMC4481717

[28] Purse BV, Mellor PS, Rogers DJ, Samuel AR, Mertens PP, Baylis M. 2005. Climate change and the recent emergence of bluetongue in Europe. Nature Reviews Microbiology, 3(2), 171–181. 15685226 10.1038/nrmicro1090

[29] Purse BV, Carpenter S, Venter GJ, Bellis G, Mullens BA. 2015. Bionomics of temperate and tropical *Culicoides* midges: knowledge gaps and consequences for transmission of *Culicoides*-borne viruses. Annual Review of Entomology, 60, 373–392. 10.1146/annurev-ento-010814-020614 25386725

[30] Van Dijk AA, Huismans H. 1982. The effect of temperature on the *in vitro* transcriptase reaction of bluetongue virus, epizootic haemorrhagic disease virus and African horsesickness virus. Onderstepoort Journal of Veterinary Research, 49(4), 227–232. 6308533

[31] Wada Y. 1999. *Culicoides* biting midges of Japan (Diptera: Ceratopogonidae). Transaction of Nagasaki Biology Society, 50, 45–71.

[32] WHO. 1961. Arthropod-borne viruses, in World Health Organisation technical report series. Anonymous, Editor. World Health Organisation: Geneva. 219 p.

[33] WHO. 1967. Arboviruses and human disease, in World Health Organisation technical report series. Anonymous, Editor. World Health Organisation: Geneva. 369 p.

[34] Wirth WW, Hubert AA. 1989. The *Culicoides* of Southeast Asia (Diptera: Ceratopogonidae). Memoirs of the American Entomological Institute, 44(1), 1–509.

[35] Wittmann EJ, Baylis M. 2000. Climate change: effects on *Culicoides*-transmitted viruses and implications for the UK. Veterinary Journal, 160(2), 107–117. 10.1053/tvjl.2000.0470 10985802

[36] Xiao L, Meng JX, Li N, Gao L, He YW, Yang H, Hu Q, Li HC, Zhu JB. 2014. Isolation and identification of bluetongue virus in 2012 in Shizong County of Yunnan Province. Chinese Journal of Animal Infectious Diseases, 22(4), 1–6.

[37] Yang H, Xiao L, Wang J, Meng J, Lv M, Liao D, Song J, Gao L, Xiong H, He Y, Niu B, Chuang X, Li H. 2017. Phylogenetic characterization genome segment 2 of Bluetongue Virus strains belonging to serotypes 5, 7 and 24 isolated for the first time in China during 2012 to 2014. Transboundary and Emerging Disease, 64(4), 1317–1321. 10.1111/tbed.12479 26865326

[38] Yu CY, Wang JS. 2001. Role of chicken serum in inhibiting *Leucocytozoon caulleryi* development in *Culicoides arakawae* infected by membrane-feeding of infective blood meals. Parasitology Research, 87(9), 698–701. 11570552 10.1007/s004360100398

[39] Zhang NZ, Zhang KL, Li ZH, Chan-yu NC, Hu YL, Li G, Zhao K, Zou FZ, Xu WZ, Li SX, Li CD, Zhang YS, Xu YS, Liu SH, Zhou XC, Dou WX, Bao CH, Zhao XQ. 1989. A Report of the Investigation and Research of Bluetongue on Sheep. Yunnan Journal of Animal Science and Veterinary Medicine, 18(4), 3–13.

[40] Zhang N, Li Z, Zhang F, Zhu J. 2004. Studies on bluetongue disease in the People’s Republic of China. Veterinaria Italiana, 40(3), 51–56. 20419635

[41] Zhao QY, Luo JC, Su Y, Zhang YJ, Tu GW, Luo Z. 2021. Propensity score matching with R: conventional methods and new features. Annals of Translational Medicine, 9(9), 812. 34268425 10.21037/atm-20-3998 PMC8246231

